# Measurement Properties of the Health Literacy Questionnaire in the Understanding Multiple Sclerosis Massive Open Online Course Cohort: A Rasch Analysis

**DOI:** 10.3928/24748307-20220720-01

**Published:** 2022-07

**Authors:** Barnabas Bessing, Cynthia A. Honan, Ingrid van der Mei, Bruce V. Taylor, Suzi B. Claflin

## Abstract

**Background:**

Online health education and other electronic health improvement strategies are developing rapidly, highlighting the growing need for valid scales to assess health literacy (HL). One comprehensive HL scale is the Health Literacy Questionnaire (HLQ), but little is known about its measurement properties in online health education cohorts.

**Objective:**

The purpose of this study was to determine if the multidimensional HLQ is an appropriate tool to measure HL in a cohort of Understanding Multiple Sclerosis (MS) online course enrollees.

**Methods:**

Participants who enrolled in the first two open enrollments of the Understanding MS online course completed the HLQ (*N* = 1,182) in an online survey prior to beginning course materials. We used Rasch analysis to assess the measurement properties of the HLQ.

**Key Results:**

The nine Domains of the HLQ each had ordered category function and a good fit with the Rasch model. Each domain was one-dimensional and exhibited good internal consistency and reliability. None of the 44 individual items of the HLQ demonstrated item bias or local dependency. However, while the overall fit was good, few measurement gaps were identified in this cohort for participants in each of the nine Domains, meaning that the HLQ may have low measurement precision in some participants.

**Conclusion:**

Our analysis of the HLQ indicated acceptable measurement properties in a cohort of Understanding MS online course enrollees. Although reliable information on nine separate constructs of HL was obtainable in the current study indicating that the HLQ can be used in similar cohorts, its limitations must be also considered. [***HLRP: Health Literacy Research and Practice*. 2022;6(3):e200–e212.**]

**Plain Language Summary:**

In this study, we have shown that the HLQ is suitable for measuring HL in an online public health educational platforms for chronic diseases including multiple sclerosis. This finding adds to the evidence that the HLQ can be widely used in measuring HL in different settings, populations, and health educational platforms.

The World Health Organization defines health literacy (HL) as “the cognitive and social skills which determine the motivation and ability of individuals to gain access to, understand and use information in ways which promote and maintain good health” (Nutbeam, 1998). HL has gained a lot of attention in recent years (Sørensen et al., 2012) because of increasing evidence demonstrating its strong association with health inequalities and health outcomes (Beauchamp et al., 2015; Berkman et al., 2011).

HL plays a vital role in achieving effective participation, and empowerment of people and communities (Nutbeam, 1998). It is also an important component of public health and a determinant of health equity. Multiple sclerosis (MS) is a chronic neurodegenerative disease where the immune system attacks and gradually impairs the function of the central nervous system (Wilkins, 2017). It has been shown that adequate HL is associated with improved self-care skills, management of symptoms, understanding and use of health information, participatory decision-making and compliance with treatments, empowers the patient/family/caregiver, and fosters patient shared decision-making for optimized collaborative care in chronic neurological diseases like MS (Chiovetti, 2006; Henson, 2016; Jafari et al., 2020; Lejbkowicz et al., 2012; Rieckmann et al., 2015). Conversely, lower levels of HL are associated with poor health outcomes and increased health care use in people living with MS (Marrie et al., 2014).

In this study, we assessed HL among enrollees in the Understanding MS online course, including members of the MS community (e.g., people living with MS, care givers) and interested laypeople, prior to beginning coursework. There are myriad HL assessment tools designed for use in a variety of study populations. Of these, we chose the Health Literacy Questionnaire (HLQ). The HLQ was developed using a comprehensive “validity-driven” approach (Osborne et al., 2013) and the tool comprises nine independent Domains with 44 total items that holistically capture different aspects of HL (Osborne et al., 2013). The HLQ has excellent psychometric properties and has been culturally adapted and/or validated in different populations, settings, and languages. For example, it has been adapted and/or validated in German, (Nolte et al., 2017), Danish (Maindal et al., 2016), Slovakian (Kolarcik et al., 2017), Dutch (Rademakers et al., 2020), and Iranian cohorts (Ahmadi & Salehi, 2019), as well as health professional university students (Mullan et al., 2017), older adults (Huang et al., 2019; Morris et al., 2017), people with metabolic and cardiovascular risk (Debussche et al., 2018; Richtering et al., 2017), and recently hospitalized patients (Jessup et al., 2017). However, despite the wide applicability of the HLQ, the suitability of an instrument may differ across settings or populations. Therefore, it is important to assess the performance of the HLQ in the population of interest before applying the instrument and interpreting scores (Osborne et al., 2013).

Rasch modelling is an approach used to evaluate the psychometric properties of self-reported health outcome scales like the HLQ (Richtering et al., 2017; Tennant & Conaghan, 2007). Although the usual item response theory (IRT) creates response models to fit the data, Rasch modeling does the reverse by predicting if observed responses fit the pattern of the Rasch model (Bond & Fox, 2015; Hendriks et al., 2012), which is a special case of IRT. Rasch model requires the identification and measurement of a single attribute at a time. The Rasch approach has several advantages, including providing valid summation of raw (ordinal) scores, category response ordering, item difficulty relative to person ability, and item bias and response dependency (Prieto et al., 2003), which are key to assessing scale validity and reliability for one-dimensionality. Here, we extend the current evidence on the applicability of the HLQ using Rasch analysis and validate the HLQ for use in a large online health education setting for the first time.

## Methods

### Ethics

This study was conducted in compliance with the Declaration of Helsinki of 1975, as revised in 2000, and was approved by the University of Tasmania's Social Science Human Research Ethics Committee (H0017924; H0018314). All participants gave their informed consent.

### Study Design and Data Collection

We have developed a freely available massive open online course (MOOC) entitled “Understanding MS.” The course presents participants with up-to-date, evidence-based information on the biology, management, and prevention of MS. The course content is described in detail elsewhere (Claflin et al., 2020). Participants in this cohort study were those who expressed interest in taking part in course-related research on their enrollment form. The research team contacted interested participants via email with details about the cohort study and a link to the surveys, project information sheet, and consent form.

Study participants completed an online survey prior to beginning course materials, including demographic questions and the 44-item HLQ. The data were de-identified at collection using course platform-generated participant ID numbers and remained so for analysis.

### The Health Literacy Questionnaire

The HLQ contains nine independent Domains, with a total of 44 items (Osborne et al., 2013) that capture different aspects of HL to assess population, group and individual HL needs. The HLQ consists of two parts containing items with differing response option formats. Part 1, comprising Domains 1–5, contains items with a 4-point Likert-type response option rating scale assessing the level of agreement from (1) *strongly disagree* to (4) *strongly agree*. Part 2, covering Domains 6–9, contains items with a 5-point Likert-type response option rating scale assessing the level of capability/ difficulty on each item from (1) *can't do or always difficult* to (5) *always easy*. The complete HLQ provides nine separate Domain scores. Each Domain score is calculated by averaging the scores of items that define each Domain. The HLQ does not provide an overall score (Osborne et al., 2013).

### Statistical Analysis

Stata 16.1 was used for data cleaning, management, and determining the descriptive statistics of the cohort. Participants were identified with a numerical user ID generated by the online course platform. Using this ID, we identified participants who completed a survey before both enrollments and only included data collected before the first enrollment. Similarly, we identified repeated responses and included the most complete survey or, if equally complete, the survey completed first. Those who did not complete all of the HLQ were also excluded. Rasch analysis was conducted using Win-steps software, version 4.5.5 (Linacre, 2019). It is a method of psychometric probability-based analysis that predicts if observed responses fit the pattern of the Rasch model (Bond & Fox, 2015), which is a special case of item response theory. If the model requirement is met, it identifies the measurement and structural properties of a scale (or instrument) including the relative difficulty of each item on the scale and maps these item difficulties against person ability levels. In this way it is possible to ascertain whether the difficulty level of items is appropriate for the assessment of individuals who have a particular level of skill (Bond & Fox, 2015). Rasch modeling is widely used to assess the psychometric properties of scales, test items and questionnaires in health and education (Bessing et al., 2021; Bond & Fox, 2015; Morris et al., 2017).

We first examined the category function for each of the nine Domains of the HLQ using the category frequencies, average measures, category fit statistics, threshold estimates, and probability curves. The diagnosis of the appropriate response category function enhances the validity and reliability of the HLQ (Bond & Fox, 2015; Cordier et al., 2018). We evaluated the category ordering of item response options by assessing whether each response option category has a minimum of 10 observations and the category average observed logit measures increased monotonically in accordance with the specified response option scale. Failure to meet this response option category ordering requirement is an indication of either poorly defined categories or inclusion of items that are not consistent with the construct being measured. We also assessed category step (threshold) ordering using the Andrich thresholds and category characteristic curves including detailed inspection of the item category distractor frequencies for each response category. Ideally, the Andrich thresholds (magnitude between categories) should increase monotonically with no overlaps or large gaps (>5 logits) between two adjacent categories. Steps (threshold) disordering could mean there are gaps, underutilized category or the category defines a narrow section of the construct being measured (Cordier et al., 2018).

The overall fit to the Rasch model expectations for each of the nine HLQ Domains was assessed in the Understanding MS course enrollees (Tennant & Conaghan, 2007). We also analyzed the fit to the Rasch model expectations of all items within each of the HLQ Domains. We determined goodness of fit for each HLQ Domain and corresponding individual items using the mean square (MNSQ) and z-standardized scores (Bessing et al., 2021; Bond & Fox, 2015). We used 0.6–1.4 MNSQ infit and outfit values as the acceptable fit range in this study, which is recommended for rating scales and surveys (Linacre, 2019).

We assessed several other psychometric properties of the HLQ Domains and items. These included one-dimensionality, internal consistency and reliability, Cronbach alpha test reliability, sex differential item functioning (DIF), response dependence, and scale/item targeting (described in **Table 1**). Although the Cronbach alpha test reliability is not important for the Rasch model, it does provide additional information about reliability according to classical test theory.

**Table 1:**
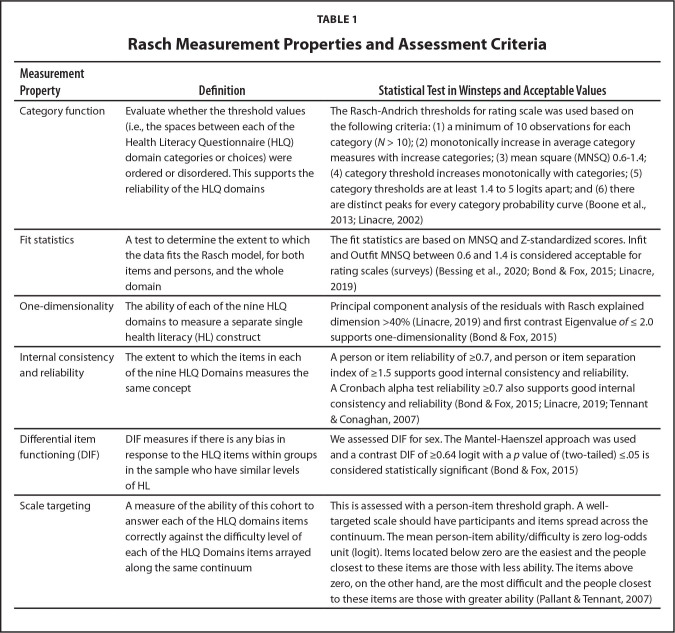
Rasch Measurement Properties and Assessment Criteria

**Measurement Property**	**Definition**	**Statistical Test in Winsteps and Acceptable Values**
Category function	Evaluate whether the threshold values (i.e., the spaces between each of the Health Literacy Questionnaire (HLQ) domain categories or choices) were ordered or disordered. This supports the reliability of the HLQ domains	The Rasch-Andrich thresholds for rating scale was used based on the following criteria: (1) a minimum of 10 observations for each category (*N* > 10); (2) monotonically increase in average category measures with increase categories; (3) mean square (MNSQ) 0.6–1.4; (4) category threshold increases monotonically with categories; (5) category thresholds are at least 1.4 to 5 logits apart; and (6) there are distinct peaks for every category probability curve (Boone et al., 2013; Linacre, 2002)
Fit statistics	A test to determine the extent to which the data fits the Rasch model, for both items and persons, and the whole domain	The fit statistics are based on MNSQ and Z-standardized scores. Infit and Outfit MNSQ between 0.6 and 1.4 is considered acceptable for rating scales (surveys) (Bessing et al., 2020; Bond & Fox, 2015; Linacre, 2019)
One-dimensionality	The ability of each of the nine HLQ domains to measure a separate single health literacy (HL) construct	Principal component analysis of the residuals with Rasch explained dimension >40% (Linacre, 2019) and first contrast Eigenvalue *of* ≤ 2.0 supports one-dimensionality (Bond & Fox, 2015)
Internal consistency and reliability	The extent to which the items in each of the nine HLQ Domains measures the same concept	A person or item reliability of ≥0.7, and person or item separation index of ≥1.5 supports good internal consistency and reliability. A Cronbach alpha test reliability ≥0.7 also supports good internal consistency and reliability (Bond & Fox, 2015; Linacre, 2019; Tennant & Conaghan, 2007)
Differential item functioning (DIF)	DIF measures if there is any bias in response to the HLQ items within groups in the sample who have similar levels of HL	We assessed DIF for sex. The Mantel-Haenszel approach was used and a contrast DIF of ≥0.64 logit with a *p* value of (two-tailed) ≤.05 is considered statistically significant (Bond & Fox, 2015)
Scale targeting	A measure of the ability of this cohort to answer each of the HLQ domains items correctly against the difficulty level of each of the HLQ Domains items arrayed along the same continuum	This is assessed with a person-item threshold graph. A well-targeted scale should have participants and items spread across the continuum. The mean person-item ability/difficulty is zero log-odds unit (logit). Items located below zero are the easiest and the people closest to these items are those with less ability. The items above zero, on the other hand, are the most difficult and the people closest to these items are those with greater ability (Pallant & Tennant, 2007)

## Results

**Figure 1.**
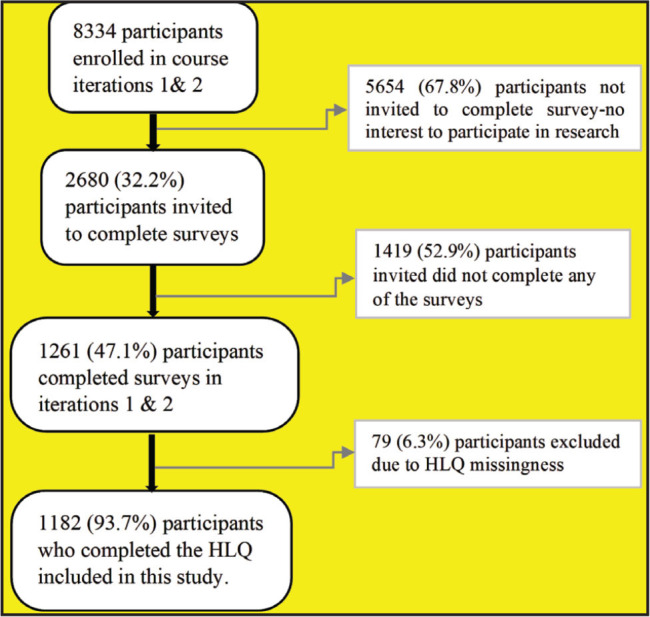
Participant's flowchart. HLQ = Health Literacy Questionnaire.

In total, 8334 people enrolled in the first two enrollments of the Understanding MS MOOC. Of these, 2,680 (32.2%) were invited to take part in the cohort studies because they indicated that they were interested in participating in research at enrollment. Of those invited, 1,261 (47.1%) participants completed the pre-course surveys in iterations 1 and 2. Of those who completed the pre-course surveys, 1182 (93.7%) have complete HLQ data for analyses (**Figure 1**).

### Participant's Characteristics

The characteristics of study participants are presented in **Table 2**. The average age of participants was 48 years, and most participants were women (86%), married or in a de facto partnership (68%) and spoke English at home (92%). Participants were highly educated, with 57% having an associate degree or higher.

**Table 2:**
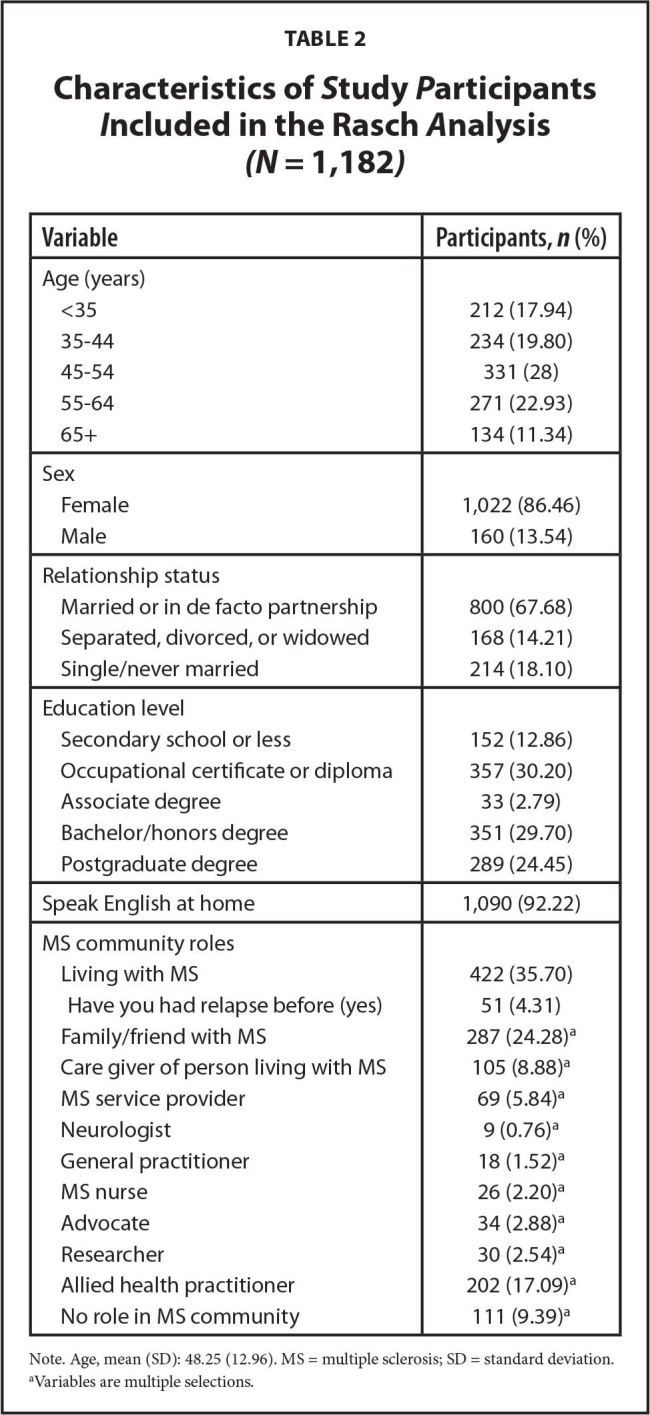
Characteristics of *S*tudy *P*articipants *I*ncluded in the Rasch *A*nalysis (*N* = 1,182)

**Variable**	**Participants, *n* (%)**

Age (years)	
<35	212 (17.94)
35–44	234 (19.80)
45–54	331 (28)
55–64	271 (22.93)
65+	134 (11.34)

Sex	
Female	1,022 (86.46)
Male	160 (13.54)

Relationship status	
Married or in de facto partnership	800 (67.68)
Separated, divorced, or widowed	168 (14.21)
Single/never married	214 (18.10)

Education level	
Secondary school or less	152 (12.86)
Occupational certificate or diploma	357 (30.20)
Associate degree	33 (2.79)
Bachelor/honors degree	351 (29.70)
Postgraduate degree	289 (24.45)

Speak English at home	1,090 (92.22)

MS community roles	
Living with MS	422 (35.70)
Have you had relapse before (yes)	51 (4.31)
Family/friend with MS	287 (24.28)^a^
Care giver of person living with MS	105 (8.88)^a^
MS service provider	69 (5.84)^a^
Neurologist	9 (0.76)^a^
General practitioner	18 (1.52)^a^
MS nurse	26 (2.20)^a^
Advocate	34 (2.88)^a^
Researcher	30 (2.54)^a^
Allied health practitioner	202 (17.09)^a^
No role in MS community	111 (9.39)^a^

Note. Age, mean (SD): 48.25 (12.96). MS = multiple sclerosis; SD = standard deviation.

a Variables are multiple selections.

### Rasch Analysis

***HLQ category function.*** When examining the category function for each of the nine HLQ Domains, there were more than ten observations per category and their average category measures increased monotonically in 4 or 5 distinct ordered response option categories depending on the Domain (**Table 3**). This indicated each of the nine HLQ Domain rating categorizations were satisfactory and well defined.

**Table 3:**
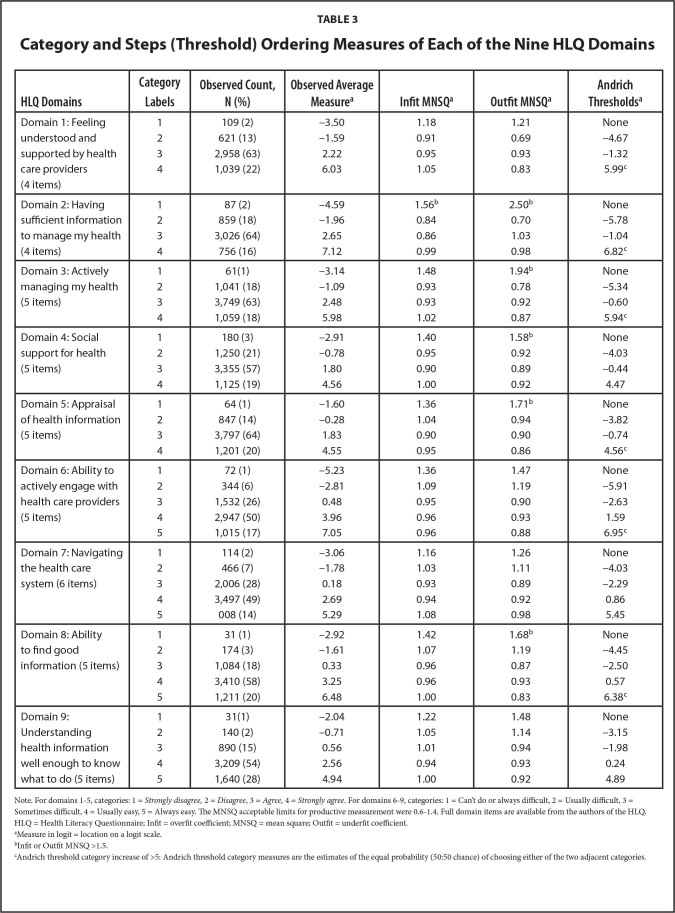
Category and Steps (Threshold) Ordering Measures of Each of the Nine HLQ Domains

**HLQ Domains**	**Category Labels**	**Observed Count, N (%)**	**Observed Average Measure^a^**	**Infit MNSQ^a^**	**Outfit MNSQ^a^**	**Andrich Thresholds^a^**

Domain 1: Feeling understood and supported by health care providers (4 items)	1	109 (2)	−3.50	1.18	1.21	None
	2	621 (13)	−1.59	0.91	0.69	−4.67
	3	2,958 (63)	2.22	0.95	0.93	−1.32
	4	1,039 (22)	6.03	1.05	0.83	5.99^c^

Domain 2: Having sufficient information to manage my health (4 items)	1	87 (2)	−4.59	1.56^b^	2.50^b^	None
	2	859 (18)	−1.96	0.84	0.70	−5.78
	3	3,026 (64)	2.65	0.86	1.03	−1.04
	4	756 (16)	7.12	0.99	0.98	6.82^c^

Domain 3: Actively managing my health (5 items)	1	61(1)	−3.14	1.48	1.94^b^	None
	2	1,041 (18)	−1.09	0.93	0.78	−5.34
	3	3,749 (63)	2.48	0.93	0.92	−0.60
	4	1,059 (18)	5.98	1.02	0.87	5.94^c^

Domain 4: Social support for health (5 items)	1	180 (3)	−2.91	1.40	1.58^b^	None
	2	1,250 (21)	−0.78	0.95	0.92	−4.03
	3	3,355 (57)	1.80	0.90	0.89	−0.44
	4	1,125 (19)	4.56	1.00	0.92	4.47

Domain 5: Appraisal of health information (5 items)	1	64 (1)	−1.60	1.36	1.71^b^	None
	2	847 (14)	−0.28	1.04	0.94	−3.82
	3	3,797 (64)	1.83	0.90	0.90	−0.74
	4	1,201 (20)	4.55	0.95	0.86	4.56^c^

Domain 6: Ability to actively engage with health care providers (5 items)	1	72 (1)	−5.23	1.36	1.47	None
	2	344 (6)	−2.81	1.09	1.19	−5.91
	3	1,532 (26)	0.48	0.95	0.90	−2.63
	4	2,947 (50)	3.96	0.96	0.93	1.59
	5	1,015 (17)	7.05	0.96	0.88	6.95^c^

Domain 7: Navigating the health care system (6 items)	1	114 (2)	−3.06	1.16	1.26	None
	2	466 (7)	−1.78	1.03	1.11	−4.03
	3	2,006 (28)	0.18	0.93	0.89	−2.29
	4	3,497 (49)	2.69	0.94	0.92	0.86
	5	008 (14)	5.29	1.08	0.98	5.45

Domain 8: Ability to find good information (5 items)	1	31 (1)	−2.92	1.42	1.68^b^	None
	2	174 (3)	−1.61	1.07	1.19	−4.45
	3	1,084 (18)	0.33	0.96	0.87	−2.50
	4	3,410 (58)	3.25	0.96	0.93	0.57
	5	1,211 (20)	6.48	1.00	0.83	6.38^c^

Domain 9: Understanding health information well enough to know what to do (5 items)	1	31(1)	−2.04	1.22	1.48	None
	2	140 (2)	−0.71	1.05	1.14	−3.15
	3	890 (15)	0.56	1.01	0.94	−1.98
	4	3,209 (54)	2.56	0.94	0.93	0.24
	5	1,640 (28)	4.94	1.00	0.92	4.89

Note. For domains 1–5, categories: 1 = *Strongly disagree,* 2 = *Disagree*, 3 = *Agree,* 4 = *Strongly agree*. For domains 6–9, categories: 1 = Can't do or always difficult, 2 = Usually difficult, 3 = Sometimes difficult, 4 = Usually easy, 5 = Always easy. The MNSQ acceptable limits for productive measurement were 0.6–1.4. Full domain items are available from the authors of the HLQ. HLQ = Health Literacy Questionnaire; Infit = overfit coefficient; MNSQ = mean square; Outfit = underfit coefficient.

a Measure in logit = location on a logit scale.

b Infit or Outfit MNSQ >1.5.

c Andrich threshold category increase of >5: Andrich threshold category measures are the estimates of the equal probability (50:50 chance) of choosing either of the two adjacent categories.

Examination of the Andrich thresholds demonstrated increased thresholds monotonically along the continuum, indicating the categories were distinct for each of the nine Domains. However, 6 of 9 Domains had Andrich threshold magnitudes >5 logits between the last two categories, indicating potential measurement gaps between item response category difficulty levels and participants' ability.

**Figure 2.**
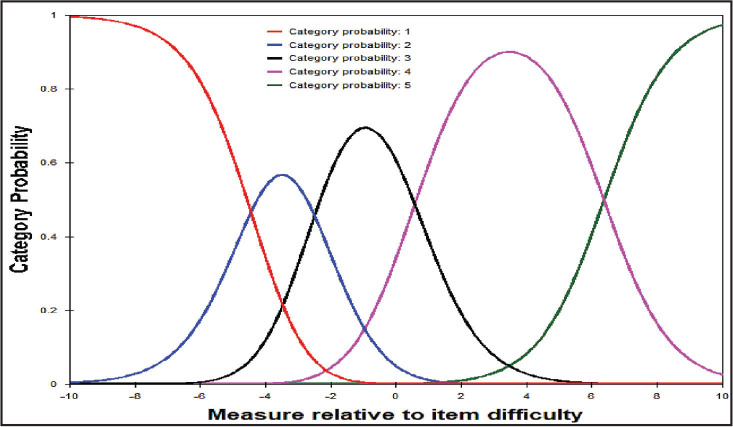
Item category characteristic curve (CCC) for the Health Literacy Questionnaire (HLQ) item 26 (domain 8): “Find information about health problems.” The CCC shows how probable the observations of each of the five categories relative to item 26 difficulty. 1 = *Can't do or always difficult*, 2 = *Usually difficult*, 3 = *Sometimes difficult*, 4 = *Usually easy*, 5 = *Always easy*. The probability of selecting each of the five categories is plotted along the vertical (y) axis. The range of person total scores for the HLQ item 26 relative to the item 26 difficulty level is plotted on the horizontal (x) axis on a logit scale. The intersection of any two adjacent categories represents the threshold peak measures—the point at which there is equal chance (50:50 probability) of choosing either of the categories.

We further examined the category probability curves of each of the nine Domains of the HLQ and respective items within each Domain. There are distinct thresholds for each response option category within each item, with each category response option also exhibiting distinct peaks on the probability curves. For example, **Figure 2** gives information about the appropriateness of the response categories for the HLQ item “Find information about health problems.” From **Figure 2**, the Y-axis (ranged 0–1) depicts the anticipated probability of each response category to be endorsed by the respondents. The X-axis stands for the item difficulty (endorsable), and a positive value means a higher ability to be endorsable and negative value means a lower ability to be endorsable.

**Figure 2** indicates that participants who showed positive attitude toward “Find information about health problems” (those with high positive values on the x-axis) were more likely to endorse higher categories (category: 5, *Always easy*). Similarly, participants who showed negative attitude toward “Find information about health problems” (those with low values on the x-axis) tend to endorse lower categories (category: 1, *Can't do or always difficult*). This trend was similarly found across all Domains and individual items of the HLQ (see **Figure A** and **Figure B** for a graphical depiction of all remaining items). Together this information indicates that, on average, participants with higher ability (agreeable) increasingly endorsed higher categories and those that were not as agreeable increasingly endorsed lower categories as expected. These suggest that the response categories in the HLQ function as intended.

**Figure A:**
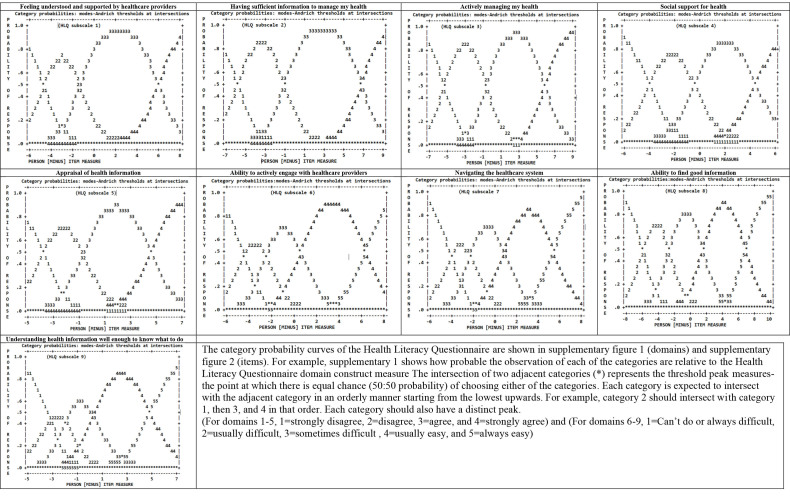
HLQ domains category probability curves

**Figure B:**
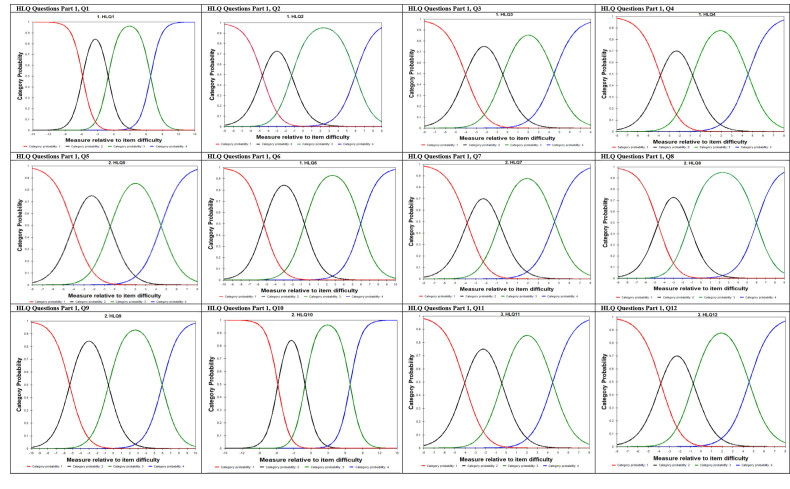

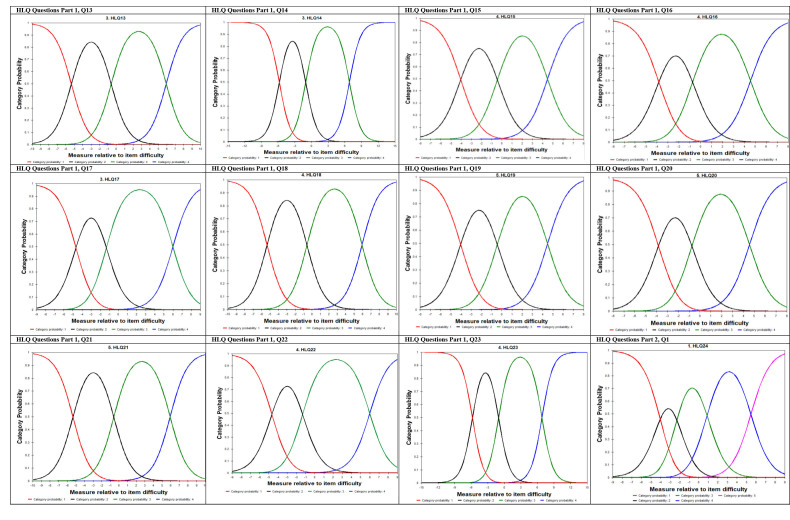

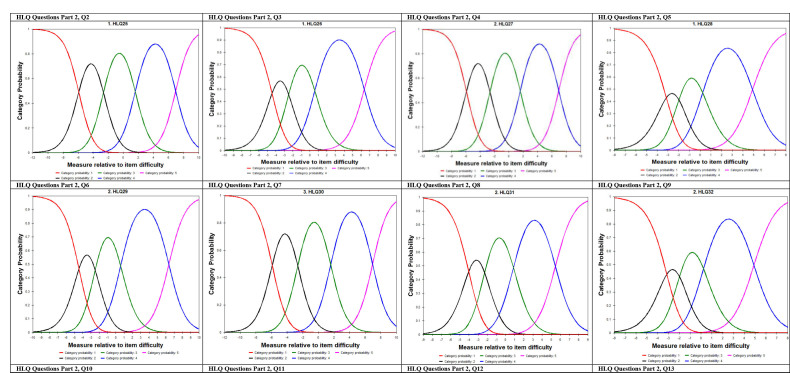

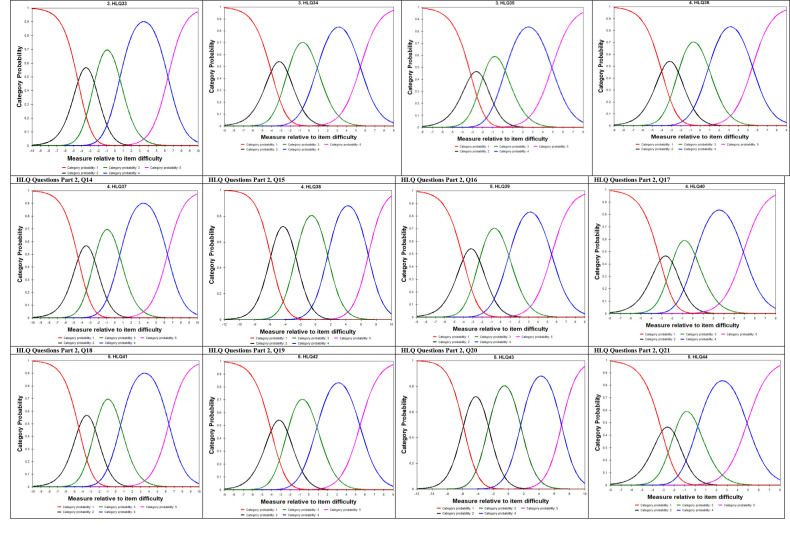
HLQ 44 items category probability curves

***Fit statistics.*** The nine Domains of the HLQ were a good fit for the Rasch model, with infit and outfit MNSQ values between 0.82 and 1.00, falling within the acceptable range of 0.6 to 1.4 (**Table 4**). All items under each HLQ Domain were scored appropriately and they functioned as expected (Linacre, 2002). The individual items were in the acceptable range for good model fit, although the item “I spend quiet a lot of time actively managing my health” (in Domain 3: Actively managing my health) was slightly underfitting with an outfit MNSQ of 1.51 (cut-off 1.40) (**Table 5**). This implies there was too much variation in the responses of participants for this item (Bond & Fox, 2015).

**Table 4:**
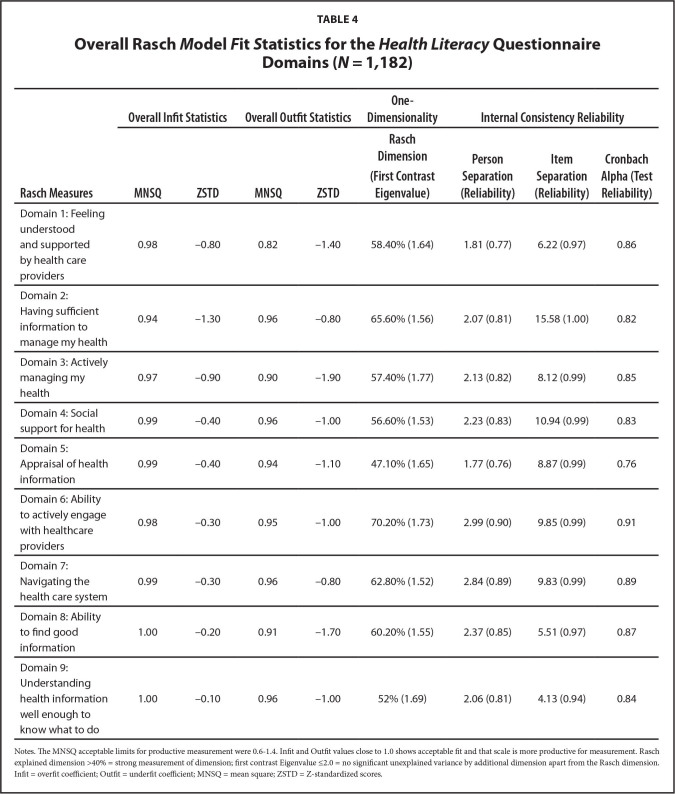
Overall Rasch *M*odel *F*it *S*tatistics for the *Health Literacy* Questionnaire Domains (*N *= 1*,*182)

**Rasch Measures**	**Overall Infit Statistics**		**Overall Outfit Statistics**		**One-Dimensionality**	**Internal Consistency Reliability**		
	**MNSQ**	**ZSTD**	**MNSQ**	**ZSTD**	**Rasch Dimension (First Contrast Eigenvalue)**	**Person Separation (Reliability)**	**Item Separation (Reliability)**	**Cronbach Alpha (Test Reliability)**
Domain 1: Feeling understood and supported by health care providers	0.98	−0.80	0.82	−1.40	58.40% (1.64)	1.81 (0.77)	6.22 (0.97)	0.86
Domain 2: Having sufficient information to manage my health	0.94	−1.30	0.96	−0.80	65.60% (1.56)	2.07 (0.81)	15.58 (1.00)	0.82
Domain 3: Actively managing my health	0.97	−0.90	0.90	−1.90	57.40% (1.77)	2.13 (0.82)	8.12 (0.99)	0.85
Domain 4: Social support for health	0.99	−0.40	0.96	−1.00	56.60% (1.53)	2.23 (0.83)	10.94 (0.99)	0.83
Domain 5: Appraisal of health information	0.99	−0.40	0.94	−1.10	47.10% (1.65)	1.77 (0.76)	8.87 (0.99)	0.76
Domain 6: Ability to actively engage with healthcare providers	0.98	−0.30	0.95	−1.00	70.20% (1.73)	2.99 (0.90)	9.85 (0.99)	0.91
Domain 7: Navigating the health care system	0.99	−0.30	0.96	−0.80	62.80% (1.52)	2.84 (0.89)	9.83 (0.99)	0.89
Domain 8: Ability to find good information	1.00	−0.20	0.91	−1.70	60.20% (1.55)	2.37 (0.85)	5.51 (0.97)	0.87
Domain 9: Understanding health information well enough to know what to do	1.00	−0.10	0.96	−1.00	52% (1.69)	2.06 (0.81)	4.13 (0.94)	0.84

Notes. The MNSQ acceptable limits for productive measurement were 0.6–1.4. Infit and Outfit values close to 1.0 shows acceptable fit and that scale is more productive for measurement. Rasch explained dimension >40% = strong measurement of dimension; first contrast Eigenvalue ≤2.0 = no significant unexplained variance by additional dimension apart from the Rasch dimension. Infit = overfit coefficient; Outfit = underfit coefficient; MNSQ = mean square; ZSTD = Z-standardized scores.

**Table 5:**
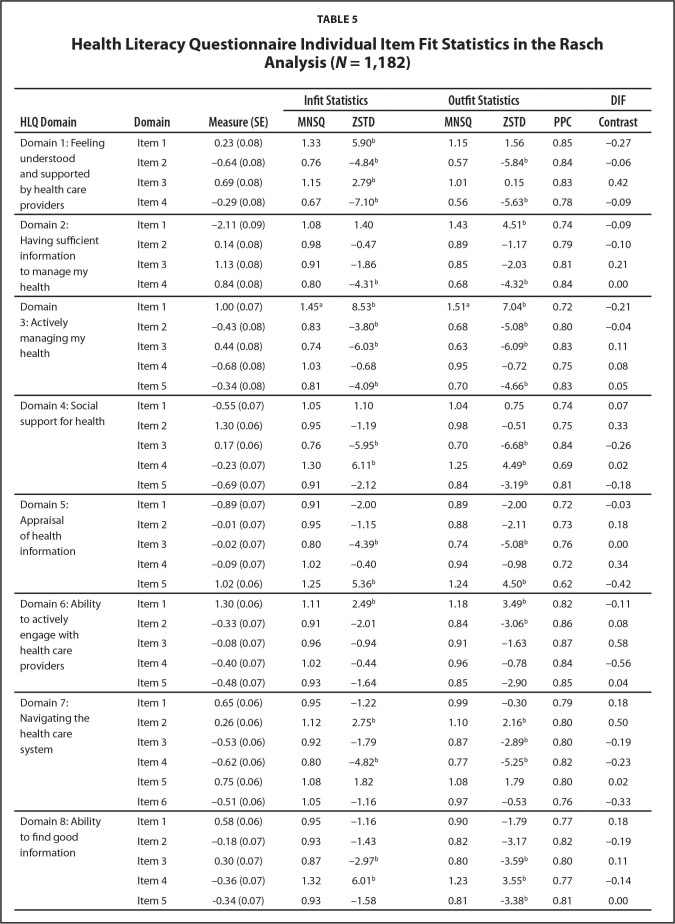

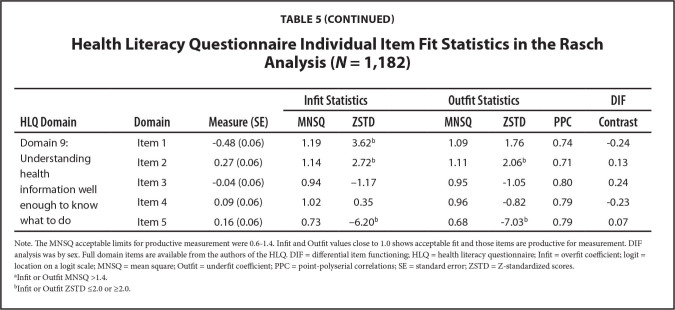
Health Literacy Questionnaire Individual Item Fit Statistics in the Rasch Analysis (*N* = 1,182)

**HLQ Domain**	**Domain**	**Measure (SE)**	**Infit Statistics**		**Outfit Statistics**			**DIF**

			**MNSQ**	**ZSTD**	**MNSQ**	**ZSTD**	**PPC**	**Contrast**

Domain 1: Feeling understood and supported by health care providers	Item 1	0.23 (0.08)	1.33	5.90^b^	1.15	1.56	0.85	−0.27
	Item 2	−0.64 (0.08)	0.76	−4.84^b^	0.57	−5.84^b^	0.84	−0.06
	Item 3	0.69 (0.08)	1.15	2.79^b^	1.01	0.15	0.83	0.42
	Item 4	−0.29 (0.08)	0.67	−7.10^b^	0.56	−5.63^b^	0.78	−0.09

Domain 2: Having sufficient information to manage my health	Item 1	−2.11 (0.09)	1.08	1.40	1.43	4.51^b^	0.74	−0.09
	Item 2	0.14 (0.08)	0.98	−0.47	0.89	−1.17	0.79	−0.10
	Item 3	1.13 (0.08)	0.91	−1.86	0.85	−2.03	0.81	0.21
	Item 4	0.84 (0.08)	0.80	−4.31^b^	0.68	−4.32^b^	0.84	0.00

Domain 3: Actively managing my health	Item 1	1.00 (0.07)	1.45^a^	8.53^b^	1.51^a^	7.04^b^	0.72	−0.21
	Item 2	−0.43 (0.08)	0.83	−3.80^b^	0.68	−5.08^b^	0.80	−0.04
	Item 3	0.44 (0.08)	0.74	−6.03^b^	0.63	−6.09^b^	0.83	0.11
	Item 4	−0.68 (0.08)	1.03	−0.68	0.95	−0.72	0.75	0.08
	Item 5	−0.34 (0.08)	0.81	−4.09^b^	0.70	−4.66^b^	0.83	0.05

Domain 4: Social support for health	Item 1	−0.55 (0.07)	1.05	1.10	1.04	0.75	0.74	0.07
	Item 2	1.30 (0.06)	0.95	−1.19	0.98	−0.51	0.75	0.33
	Item 3	0.17 (0.06)	0.76	−5.95^b^	0.70	−6.68^b^	0.84	−0.26
	Item 4	−0.23 (0.07)	1.30	6.11^b^	1.25	4.49^b^	0.69	0.02
	Item 5	−0.69 (0.07)	0.91	−2.12	0.84	−3.19^b^	0.81	−0.18

Domain 5: Appraisal of health information	Item 1	−0.89 (0.07)	0.91	−2.00	0.89	−2.00	0.72	−0.03
	Item 2	−0.01 (0.07)	0.95	−1.15	0.88	−2.11	0.73	0.18
	Item 3	−0.02 (0.07)	0.80	−4.39^b^	0.74	−5.08^b^	0.76	0.00
	Item 4	−0.09 (0.07)	1.02	−0.40	0.94	−0.98	0.72	0.34
	Item 5	1.02 (0.06)	1.25	5.36^b^	1.24	4.50^b^	0.62	−0.42

Domain 6: Ability to actively engage with health care providers	Item 1	1.30 (0.06)	1.11	2.49^b^	1.18	3.49^b^	0.82	−0.11
	Item 2	−0.33 (0.07)	0.91	−2.01	0.84	−3.06^b^	0.86	0.08
	Item 3	−0.08 (0.07)	0.96	−0.94	0.91	−1.63	0.87	0.58
	Item 4	−0.40 (0.07)	1.02	−0.44	0.96	−0.78	0.84	−0.56
	Item 5	−0.48 (0.07)	0.93	−1.64	0.85	−2.90	0.85	0.04

Domain 7: Navigating the health care system	Item 1	0.65 (0.06)	0.95	−1.22	0.99	−0.30	0.79	0.18
	Item 2	0.26 (0.06)	1.12	2.75^b^	1.10	2.16^b^	0.80	0.50
	Item 3	−0.53 (0.06)	0.92	−1.79	0.87	−2.89^b^	0.80	−0.19
	Item 4	−0.62 (0.06)	0.80	−4.82^b^	0.77	−5.25^b^	0.82	−0.23
	Item 5	0.75 (0.06)	1.08	1.82	1.08	1.79	0.80	0.02
	Item 6	−0.51 (0.06)	1.05	−1.16	0.97	−0.53	0.76	−0.33

Domain 8: Ability to find good information	Item 1	0.58 (0.06)	0.95	−1.16	0.90	−1.79	0.77	0.18
	Item 2	−0.18 (0.07)	0.93	−1.43	0.82	−3.17	0.82	−0.19
	Item 3	0.30 (0.07)	0.87	−2.97^b^	0.80	−3.59^b^	0.80	0.11
	Item 4	−0.36 (0.07)	1.32	6.01^b^	1.23	3.55^b^	0.77	−0.14
	Item 5	−0.34 (0.07)	0.93	−1.58	0.81	−3.38^b^	0.81	0.00

Domain 9: Understanding health information well enough to know what to do	Item 1	−0.48 (0.06)	1.19	3.62^b^	1.09	1.76	0.74	−0.24
	Item 2	0.27 (0.06)	1.14	2.72^b^	1.11	2.06^b^	0.71	0.13
	Item 3	−0.04 (0.06)	0.94	−1.17	0.95	−1.05	0.80	0.24
	Item 4	0.09 (0.06)	1.02	0.35	0.96	−0.82	0.79	−0.23
	Item 5	0.16 (0.06)	0.73	−6.20^b^	0.68	−7.03^b^	0.79	0.07

Note. The MNSQ acceptable limits for productive measurement were 0.6–1.4. Infit and Outfit values close to 1.0 shows acceptable fit and those items are productive for measurement. DIF analysis was by sex. Full domain items are available from the authors of the HLQ. DIF = differential item functioning; HLQ = health literacy questionnaire; Infit = overfit coefficient; logit = location on a logit scale; MNSQ = mean square; Outfit = underfit coefficient; PPC = point-polyserial correlations; SE = standard error; ZSTD = Z-standardized scores.

a Infit or Outfit MNSQ >1.4.

b Infit or Outfit ZSTD ≤2.0 or ≥2.0.

***One-dimensionality. ***The PCA of the residuals for each of the nine HLQ Domains supported one-dimensionality of each model (**Table 5**). The Rasch dimension in each Domain explained >47% of the variance in the data. Explanations of >40% variance are considered strong measurements of dimension (Linacre, 2019). The total unexplained variance in each Domain had first contrast eigenvalues <2. This implied there was no significant second dimension after extracting the Rasch dimension and that the unexplained variances in each Domain were mainly due to random noise.

***Reliability and internal consistency of the HLQ.*** The person separation of ≥2.0 and person reliability of ≥0.8 for each of the nine HLQ Domains suggested that the items contained within each Domain were sensitive enough to differentiate between at least two-person ability levels (low and high) (see **Table 4**). The item separation of >4 and item reliability of >0.9 observed for each HLQ Domain were above the recommended values (separation ≥3 and reliability ≥0.9) (Cordier et al., 2018; Linacre, 2010). This indicated that our sample was large enough to confirm at least three item difficulty levels (low, medium, and high) in each HLQ Domain, supporting the construct validity of the HLQ (Linacre, 2010).

***HLQ differential item functioning.*** The DIF contrast values for items in each HLQ Domain were <0.64, offering no evidence of item bias (see **Table 5**). This indicated that participants with the same level of HL within each Domain responded consistently to the items in that Domain irrespective of their sex.

**Figure 3.**
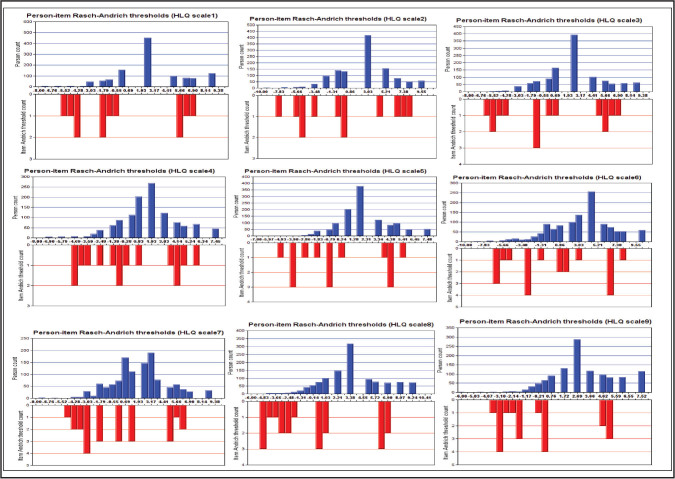
Person-item threshold distribution graph for each of the nine Health Literacy Questionnaire (HLQ) Domains. The horizontal axis is the relative location of person ability (blue) or item difficulty (red) level on a log-odds unit (logit) scale. The vertical axis is a count of people with a particular ability level (blue) or items of a particular difficulty level (red). The mean location is zero. Items (red) or people (blue) located below zero are considered less difficult items or to have lower ability level, respectively. Items or people located above zero are considered more difficult items or to have a higher ability level. These graphs provide evidence of a good overall match between person ability and item difficulty with some measurement gaps.

***Item targeting. ***The person-item Rasch-Andrich threshold distribution for each of the nine HLQ Domains on a log-odds unit (logit) scale are shown in **Figure 3**. The Domain difficulty (endorsable) levels ranged from −6.0 to 8 logits and the person ability (agreeable) levels ranged from −10 to 10. This indicated that the HLQ Domain difficulty (endorsable) levels cover most of the ability (agreeable) levels of the separate HL constructs among our participants.

However, each of the nine HLQ Domains contain gaps between participants self-reported ability and Domain difficulty levels which does not support adequate scale targeting. For example, in **Figure 3**, the HLQ Domain 4 (HLQ scale 4) items difficulty level did not cover participants with ability below −4.7 logits hence these participants self-reported these items too difficult to endorse. Similarly, there was no item difficulty matching participants with ability level between 1 and 3 logits meaning these participants self-reported these items below their ability too easy and those above their ability too difficult to endorse. It is important to note this is self-reported and not a test-based measurement and may not reflect the true ability and difficulty levels as indicated.

## Discussion

The main purpose of this article was to assess the appropriateness of the HLQ to measure HL among Understanding MS online course participants and to provide evidence that the HLQ may be used in online health education settings. Rasch analysis was specifically used to rigorously assess the HLQ's psychometric properties. We found that the HLQ is an appropriate tool for the assessment of the nine separate HL constructs in Understanding MS online course enrollees, comprising of both MS community members and interested laypeople. The nine Domains of the HLQ had no disordered response option categories and demonstrated adequate fit with the Rasch measurement model. Each of the HLQ Domains showed one-dimensionality, moderate-to-high internal consistency and reliability, no item bias and adequate scale targeting. However, there were few measurement gaps between participants ability (agreeable) and item difficulty (endorsable) levels.

In the study cohort, the nine Domains of the HLQ each had adequate ordered rating-scale categories with enough separation between them and ordered Rasch-Andrich threshold measures. The 4-point and 5-point rating scale response options used in the HLQ Domains functioned appropriately in our sample. Because of this, we maintained these rating options for the assessment of HL in our cohort.

The result of this study is consistent with previous work that found the HLQ to have good measurement properties with robust construct validity and reliability for each of its nine Domains (Ahmadi & Salehi, 2019; Huang et al., 2019; Kolarcik et al., 2017; Maindal et al., 2016; Morris et al., 2017; Nolte et al., 2017; Osborne et al., 2013; Richtering et al., 2017). This indicates that the nine HLQ domains each consistently measure a single HL construct and together they provide reliable and valid information on nine distinct HL constructs. Our work suggests that the HLQ provides fair assessments of individual HL levels across sex groups in the Understanding MS online course cohort, as is expected of a good measurement tool (Tennant & Conaghan, 2007). This suggests that sex differences found in previous studies are likely to be true differences and not measurement artifact (Maindal et al., 2016).

In the Understanding MS online course cohort, the HLQ Domains were unable to adequately distinguish all participant ability levels in HL. This was evidenced by varied gaps between participant ability and item difficulty levels. This finding that the HLQ has inadequate participant ability targeting suggests that the HLQ measurement precision is reduced for participants with very low and low HL and for those with moderate or high HL in the study cohort. This finding is similar to the results of two previous validation studies evaluating cohorts with similarly high mean educational attainment or socioeconomic status (Morris et al., 2017; Richtering et al., 2017). In these prior studies, the HLQ was found to have inadequate targeting for participants with high HL among older adults who presented to the emergency department after a fall (Morris et al., 2017) and in a population with moderate-to-high cardiovascular risk (Richtering et al., 2017). Despite these few measurement gaps in the HLQ Domains, our data using the Rasch model validated the use of the HLQ to assess HL in this cohort. It is encouraging to find the HLQ a suitable choice of instrument for use in this cohort considering that our study was overpowered and is more likely to find statistically significant problems that are not clinically significant or relevant to public health.

We have shown that the HLQ can be used to measure HL in online health educational platforms. This adds to the existing knowledge on the validated modes of delivery for HL measurements such as self-administered paper-based, online/web-based, face-to-face, and telephone-based interviews that have been explored in different settings and populations (Ahmadi & Salehi, 2019; Debussche et al., 2018; Huang et al., 2019; Jessup et al., 2017; Kolarcik et al., 2017; Maindal et al., 2016; Morris et al., 2017; Mullan et al., 2017; Nolte et al., 2017; Rademakers et al., 2020; Richtering et al., 2017).

## Strengths, Limitations, and Future Research

The major strength of this study is the use of the Rasch modeling approach to provide a rigorous psychometric assessment of the HLQ in a large cohort of online learners, which included both members of the MS community and the general public. This study had limitations. Our study had a moderate participation rate (44.1% of invited participants), which is typical of online surveys. This may have introduced nonresponse selection bias hence our findings should be interpreted with caution. A small proportion of the study cohort were men (13.5%). Although this is a common issue among MS-related cohorts, given that MS affects nearly three times as many women compared to men (Shull et al., 2020), it suggests that the results of the DIF analysis assessing the influence of sex should be interpreted with caution. The high education level of our cohort may have resulted in the observed gaps in the domain targeting. Future research should examine changes in HL in this cohort and test the sensitivity of the HLQ Domains to change over time.

## Conclusion

Here we present a robust psychometric validation of the HLQ in a large cohort in an online health education setting. The strong psychometric properties demonstrated by the HLQ in this study indicate that it is an appropriate tool for the assessment of HL among participants in the Understanding MS online course and similar settings.
